# Headache in an emergency room in Brazil

**DOI:** 10.1590/S1516-31802000000300002

**Published:** 2000-05-02

**Authors:** Marcelo Bigal, Carlos Alberto Bordini, José Geraldo Speciali

**Keywords:** Headache, General practioners, Emergency room, Cefaléia, Clínicos gerais, Unidade de emergência

## Abstract

**CONTEXT:**

When experiencing a headache attack, Brazilian patients usually look for a primary care service, where they are seen by general clinicians. In the town of Ribeirão Preto, these clinicians routinely refer patients to the Emergency Room of the University Hospital.

**OBJECTIVE:**

The objective of this study was to evaluate the quality of primary care by analyzing retrospectively the medical records of patients with a complaint of headache seen in this emergency room during the year of 1996.

**DESIGN:**

retrospective study.

**SETTIING:**

Emergency Room of the Universital Hospital, Ribeirão Preto, São Paulo, reference unit.

**PARTICIPANTS:**

1254 patients. The patients who sought the Emergency Room (ER) of the University Hospital of Ribeirão Preto, during the year of 1996 with a complaint of headache were studied retrospectively.

**MAIN MEASUREMENTS:**

Etiology, age, diagnosis, secondary cause, laboratory tests.

**RESULTS:**

Of the 1254 patients seen (61% women), 1190 (94.9%) were discharged after the administration of parenteral analgesics before they had spent 12 hours in the room. Only 64 (5.1%) patients remained for more than 12 hours. Of the patients who spent less than 12 hours in the room, 71.5% had migraine or tension type headache and did not require subsidiary exams for diagnosis. Of the patients who spent more than 12 hours in the room, 70.3% had secondary headaches.

**CONCLUSIONS:**

We conclude the primary care for headache is unsatisfactory in the Ribeirão Preto region. Many patients with primary headache are referred to tertiary care services, indicating the need for the dissemination of the diagnostic criteria of the International Headache Society to general practitioners.

## resumo

**CONTEXTO:**

Quando em crise de cefaléia, pacientes brasileiros geralmente dirigem-se a uma unidade básica de saúde, onde são atendidos por clínicos gerais. Na cidade de Ribeirão Preto, os clínicos rotineiramente referem os pacientes para a Unidade de Emergência (UE) do Hospital das Clínicas.

**OBJETIVO:**

Avaliar a qualidade do serviço primário, analisando retrospectivamente o prontuário de pacientes que procuraram a EU, no ano de 1996, com queixa de cefaléia.

**TIPO DE ESTUDO:**

Estudo retrospectivo.

**LOCAL:**

Unidade de Emergência do Hospital das Clínicas da Faculdade de Medicina de Ribeirão Preto.

**PARTICIPANTES:**

1.254 pacientes. Os pacientes da Unidade de Emergência do hospital da Universidade de Ribeirão Preto durante o ano de 1996 com crise de cefaléia foram estudados retrospectivamente.

**VARIÁVEIS ESTUDADAS:**

Etiologia, idade, diagnóstico, causa secundária e testes laboratoriais.

**RESULTADOS:**

Dos 1254 pacientes vistos (61% mulheres), 1190 (94,9%) tiveram alta, após a administração de analgésicos parenterais, com menos de 12 horas da admissão. Apenas 64 pacientes (5,1%) permaneceram mais de 12 horas. Dos que ficaram menos de 12 horas, 71,5% apresentavam migrânea ou cefaléia tipo tensional e não necessitaram exames subsidiários. Dos que permaneceram mais de 12 horas, 70,3% apresentavam cefaléias secundárias.

**CONCLUSÃO:**

Concluímos que o serviço primário é ineficaz, para a cefaléia, na região de Ribeirão Preto. Muitos pacientes com cefaléia primária são referidos para centros terciários, indicando a necessidade urgente da divulgação dos critérios diagnósticos da Sociedade Internacional de Cefaléia para clínicos gerais.

## INTRODUCTION

Headache is one the symptoms most frequently reported in doctors’ offices, involving considerable economic losses, as well as an important worsening of the quality of life of those who suffer from it.^1^ It is the most frequent cause of adult worker absenteeism in the United States.^2,3^ Despite its high incidence, headache has been little studied in Brazil. We have no data about the percentage of Brazilians who look for medical care either during an attack or between attacks. As is also the case for other countries, self-medication or looking for a pharmacy clerk is common during the acute phase.^4^

An undetermined percentage of persons with headache looks for primary care provided by general practitioners in primary care units. The cases of acute headache seen at these units which are refractory to treatment or raise doubts about their primary etiology are referred to more differentiated care units.^5,6^ This is also a routine occurrence in the town of Ribeirão Preto, where the present study was conducted.

In the present investigation we studied retrospectively the patients with a complaint of headache who sought the Emergecy Room of the University Hospital of Ribeirão Preto, a tertiary care unit, in the year of 1996, with the objective of evaluating the quality of primary care for acute headaches.

## METHODS

The patients who sought the Emergency Room (ER) of the University Hospital of Ribeirão Preto, during the year of 1996 with a complaint of headache were studied retrospectively. Since the hospital is a reference unit it receives patients from an extensive region which includes not only the municipality (population of approximately 450,000), but also towns located more than 200 km from Ribeirão Preto. Patients seen at primary health care units are referred to this unit when they are refractory to treatment or when doubts exist about the primary etiology of their complaint. As they arrive at the ER, the patients are seen by the Neurology team which consists of three residents and a supervisor with specialization in Neurology. A detailed clinical-neurological examination is performed and the patients receive parenteral analgesics or anti-inflammatory agents.

A total of 1254 patients arrived at the ER with a complaint of headache in the year of 1996. Of these, 1190 were discharged before 12 hours of permanence on the basis of a significant improvement or absence of headache and of normal clinical-neurological and subsidiary exams. The patients who spend more than 12 hours in the observation rooms are considered to have been hospitalized by the statistics service of the hospital.

The present series consists of a random sample of 165 non-hospitalized patients and of all the hospitalized patients (N = 64).

Headache was classified into 3 groups according to etiology: 1) primary headache (with the pain episode fulfilling IHS7 criteria for primary headache), 2) headache secondary to neurological disorders, and 3) headache secondary to systemic disorders. The patients were then studied in terms of clinical and epidemiological aspects and submitted to laboratory tests.

## RESULTS

In 1996, 1254 patients were referred to the ER with a complaint of headache. Of these, 769 were women (61%) and 485 were men (39%). Most patients (94.9%) spent less than 12 hours in the ER. Only 64 patients (5.1%) were hospitalized, i.e., they spent more than 12 hours in the hospital environment. Patient distribution by age is given in Table 1.

**Table 1:** Distribution by age of non-hospitalized and hospitalized patients

Age(years)	Total group of patients	Hospitalized Patients
0 - 9	127 (10.1)	14 (21.9)
10 - 19	251 (20.0)	11 (17.2)
20 - 29	391 (31.2)	12 (18.8)
30 - 39	231 (18.4)	8 (12.5)
40 - 49	144 (11.5)	7 (10.9)
50 and more	110 (8.8)	12 (18.7)
**Total**	**1254 (100)**	**64 (100)**

% given in parenthesis.

About 80% of patients were less than 40 years old. The proportion of hospitalized patients was higher among subjects either younger than 10 years or older than 50 years. The age range that least required hospitalization was 40-50 years. Table 2 shows the etiologies of the headaches presented by the patients of the present series.

**Table 2:** Distribution by etiology of the headaches presented by non-hospitalizedand hospitalized patients

	Non-hospitalized	Hospitalized
Primary headache	127 (77.0)	19 (29.7)
Headache secondary to neurological disorders	15 (9.1)	33 (51.5)
Headache secondary to systemic disorders	23 (13.9)	12 (18.8)
**Total**	**165 (100)**	**64 (100)**

*Random samples, **Total number of hospitalized patients; % given in parenthesis.

**Figure 1:**
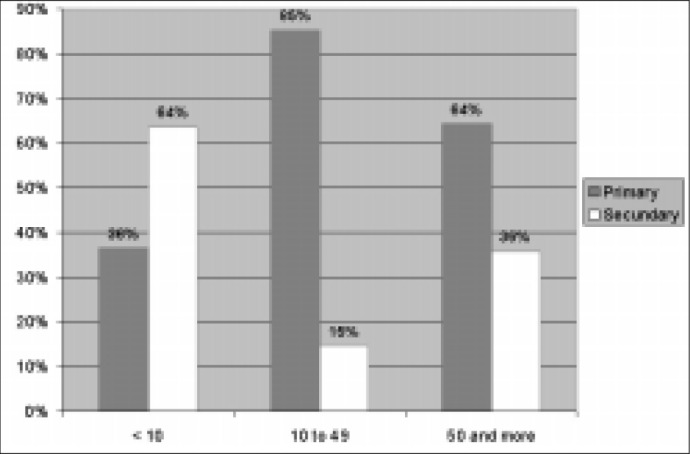
Etiologies of the headaches according to age range among non-hospitalized patients.

Primary headaches predominated among non-hospitalized patients (77.0%), whereas the percentage of headaches secondary to neurological disorders was higher among patients who required hospitalization (51.5%). However, this proportion varied widely with age range, as shown in Figure 1.

The various types of primary headaches detected in the present series of patients, according to the IHS classification, are listed in Table 3. It can be seen that 77% of the non-hospitalized patients (Table 2) had primary headache, with 56.4% of the total presenting migraine (Table 3). If we add this value to that obtained for tension headache (15.1%), we can see that 71.5% of the patients seen in the ER of a tertiary care unitt did not require hospitalization and presented migraine or tension-type headache.

**Table 3:** Primary headaches diagnosed in hospitalized and non-hospitalized patients

			Non-hospitalized	Hospitalized
Migraine			93 (56.4)	14 (21.9)
	Migraine without aura		62 (37.6)	8 (12.5)
	Migraine with aura		27 (16.3)	5 (7.8)
		Typical aura	23 (13.9)	3 (4.7)
		Basilar	4 (2.4)	2 (3.1)
	Migraine complications		4 (2.4)	1 (1.6)
		Status migranosus	3 (1.8)	1 (1.6)
		Migraine infarction	1 (0.6)	0 (0)
Tension-type			25 (15.1)	2 (3.1)
Cervicogenic			2 (1.2)	1 (1.6)
Neuralgia			1 (0.6)	0 (0)
Chronic daily			2 (1.2)	0 (0)
Conversive			3 (1.8)	2 (3.1)
Benign, due to effort			1 (0.6)	0 (0)
**Total**			**127 (77.0)**	**19 (29.7)**

% given in parenthesis.

The etiologies of the headaches secondary to neurological disorders are presented in Table 4. These headaches corresponded to less than 10% of the cases of headache that did not require patient hospitalization and even in these cases the etiology was relatively benign (Table 4). Of the hospitalized patients, 51.5% had headaches secondary to neurological disorders.

**Table 4:** Causes of headache secondary to neurological disorders among non-hospitalized and hospitalized patients

	Non-hospitalized	Hospitalized
Pseudotumor cerebri	0 (0)	2 (3.1)
Malfunctioning VPS	0 (0)	2 (3.1)
Meningitis	0 (0)	4 (6.2)
Neoplasia	0 (0)	1 (1.6)
Granuloma	0 (0)	2 (3.1)
Acute post-traumatic	5 (3.0)	5 (7.8)
Postconvulsion	2 (1.2)	3 (4.7)
Post-spinal tap	4 (2.4)	2 (3.1)
Stroke	0 (0)	4 (6.2)
Otitis	2 (1.2)	0 (0)
Subdural hematoma	0 (0)	3 (4.7)
Subarachnoid hemorrhage	0 (0)	1 (1.6)
Glaucoma	1 (0.6)	1 (1.6)
Hydrochephaly	1 (0.6)	1 (1.6)
Arteritis	0 (0)	1 (1.6)
Hygroma	0 (0)	1 (1.6)
**Total**	**15 (9.1)**	**33 (51.5)**

VPS = Ventriculoperitoneal shunt; % given in parenthesis.

Headaches secondary to systemic disorders were diagnosed in 13.9% of non-hospitalized patients and in 18.7% of hospitalized patients. The causes detected are listed in Table 5.

**Table 5:** Causes of headaches secondary to systemic disorders among non-hospitalized and hospitalized patients

	Non-hospitalized	Hospitalized
Sinusitis	8 (4.8)	5 (7.8)
Systemic infection	6 (3.6)	5 (7.8)
Drug abuse	4 (2.4)	0 (0)
Arterial hypertension	2 (1.2)	0 (0)
SHDP	0 (0)	2 (3.1)
Postural hypotension	2 (1.2)	0 (0)
Hypoglycemia	1 (0.6)	0 (0)
**Total**	**23 (17.6)**	**12 (18.8)**

SHDP = Specific hypertensive disease of pregnancy; % given in parenthesis.

The laboratory tests requested are listed in Table 6. Since the ER is not equipped with a magnetic resonance apparatus, patients who require the exam must be referred to the University Hospital on the University Campus, located about 10 km from the ER. Blood tests included blood counts, serology, biochemistry and all remaining procedures.

**Table 6:** Laboratory tests requested

	Non-hospitalized	Hospitalized
Blood tests	8 (4.8)	27 (42.2)
Skull/face radiography	21 (12.7)	16 (25.0)
Computer tomography	15 (9.1)	33 (51.6)
Spinal tap	2 (1.2)	25 (39.0)
Nuclear magnetic resonance	0 (0)	3 (4.7)
Encephalic panangiography	0 (0)	4 (6.2)
Carotid ultrasound	0 (0)	2 (3.1)
Electroencephalogram	1 (0.6)	4 (6.2)
Others	0 (0)	1 (1.6)
**Total number of patients**	**29 (17.6)**	**48 (75.0)**

% given in parenthesis.

It can be seen that for non-hospitalized patients the diagnosis was eminently clinical. The exam most frequently requested was a skull and/or face X-ray. Less than 10% of patients were submitted to computer tomography and only 1.2% were submitted to spinal taps. In contrast, 75% of hospitalized patients were submitted to exams, 56.3% to computer tomography or nuclear magnetic resonance and 39% to spinal taps.

## DISCUSSION

Several studies have reported the high incidence of headache in the population and in health services.^8–16^ Data obtained by one of us (M.E.B.) have shown that from 6006 patients were seen at two primary care units in Brazil over a period of 8 months, headache accounted for 9,3% of unscheduled visits, i.e., visits due to acute problems.^17^ General practitioners examine these patients and, when they deem it necessary they refer the patients to more differentiated health care units. The major tertiary care unit in Ribeirão Preto is the ER, where 1254 patients were seen in 1996.

Approximately 80% of the patients seen in the ER were younger than 40 years, indicating a lower incidence of headache among older people. Age and sex distribution show a predominance of headache among women and in younger ages. A larger number of hospitalizations was indicated for patients younger than 10 or older than 50 years. A greater percentage of headaches secondary to systemic disorders was observed in these age ranges. These findings are similar to those found by others.^2,9–12,16–18^

A complete work-up was performed on 17.6% of non-hospitalized patients and on 75% of hospitalized patients. Among non-hospitalized patients, 77% received a diagnosis of primary headache and among the hospitalized ones 70.3% received a diagnosis of secondary headache (Table 2). These data suggest that when the pain persists after several hours and after the administration of analgesics, neurological exams should be repeated and specialized tests such as CT scans, magnetic nuclear resonance and/or spinal tap should be carried over since there is an increased possibility of the presence of secondary headache. Moreover, these data might serve as the basis for an organized clinical reasoning at a time of cost rationalization.

In the evaluation of the medical performance of general clinicians at primary health care centers two facts were particular outstanding: 1) a small number (5.1%) of the patients referred to the ER required hospitalization, and 2) 71.5% of these patients had primary headaches that responded well to symptomatic treatment of the pain and were discharged after this procedure. This being a tertiary care unit, a higher percentage of secondary headaches was expected to be diagnosed.

The above data, taken as a whole, show that most of the patients with primary headaches were young and did not require complementary tests, i.e., they presented disorders that could have been resolved at the primary care units. Referral of patients with benign disease to a tertiary care unit raises concerns about costs and efficiency, a much debated topic in developed countries. In developing countries this fact acquires even greater importance because of the lower structuring of the health system, the precarious state investments and the low economic level of the underprivileged population. The system is insufficient to guarantee quality care in view of the high demand. When a patient is improperly referred, costly and unnecessary consequences occur: mobilization of transport with a specialized vehicle and personnel (ambulance), the hospital receiving the patient often works under conditions of overcrowding, with aggravation of the lack of beds and physicians for more serious cases. Improper referral causes a greater delay in the resolution of the problem, with a consequent prolongation of patient suffering and separation from his work, his family or his well-deserved rest. In addition, operational costs are greatly increased.

## CONCLUSIONS

We conclude that the resolution power of the primary health care system in the Ribeirão Preto region in terms of the headache symptom is very low. It should be pointed out that this region is considered to be one of the best medical centers by Brazilian doctors. Thus, it can be seen that headache, as well as precordial pain, frightens general practitioners, generating insecurity among non-specialists, with consequent diagnostic difficulties. However, in contrast to precordial pain, well-established criteria (IHS) are available for headache and in most cases a diagnosis can be made without the use of laboratory tests. Thus, there is a pressing need for a more aggressive dissemination of the diagnostic criteria of the HIS, which would lead to more space for more serious cases in tertiary care units, with decreased operational and individual costs.
